# Large Gradient High Magnetic Fields Affect Osteoblast Ultrastructure and Function by Disrupting Collagen I or Fibronectin/αβ1 Integrin

**DOI:** 10.1371/journal.pone.0051036

**Published:** 2013-01-29

**Authors:** Ai-Rong Qian, Xiang Gao, Wei Zhang, Jing-Bao Li, Yang Wang, Sheng-Meng Di, Li-Fang Hu, Peng Shang

**Affiliations:** Key Laboratory for Space Biosciences & Biotechnology, Institute of Special Environmental Biophysics, School of Life Sciences, Northwestern Polytechnical University Xi’an, Xi’an, China; Georgia Health Sciences University, United States of America

## Abstract

The superconducting magnet generates a field and field gradient product that can levitate diamagnetic materials. In this study a specially designed superconducting magnet with a large gradient high magnetic field (LG-HMF), which can provide three apparent gravity levels (μ-g, 1-g, and 2-g), was used to simulate a space-like gravity environment. The effects of LG-HMF on the ultrastructure and function of osteoblast-like cells (MG-63 and MC3T3-E1) and the underlying mechanism were investigated by transmission electromicroscopy (TEM), MTT, and cell western (ICW) assays. Under LG-HMF significant morphologic changes in osteoblast-like cells occurred, including expansion of endoplasmic reticulum and mitochondria, an increased number of lysosomes, distorted microvilli, and aggregates of actin filaments. Compared to controls, cell viability and alkaline phosphatase (ALP) secretion were significantly increased, and collagen I (col I), fibronectin (FN), vinculin, integrin α3, αv, and β1 expression were changed under LG-HMF conditions. In conclusion, LG-HMF affects osteoblast ultrastructure, cell viability, and ALP secretion, and the changes caused by LG-HMF may be related to disrupting col I or FN/αβ1 integrin.

## Introduction

The Earth’s natural geomagnetic field is perceived by some animals and used for navigation. Recent technologic innovations have led to the generation of man-made static magnetic fields up to 10 Tesla (T). Man-made high-magnetic fields (HMFs) are one of the most powerful tools for studying the properties of materials because HMFs couple directly to the electronic charge and magnetic moments of protons, neutrons, and electrons [1]. HMFs produced by a superconducting magnet have been widely used in research and medical applications, such as magnetic resonance imaging (MRI), which provides three-dimensional images of the brain and other soft tissues. Therefore, scanned patients and machine operators can be exposed to HMFs. Recently, several studies on the biological effects of man-made HMFs at the cellular level have been reported [2], [3]. It has been shown that cell exposure to HMFs (>10 T and 15 T) affects the cell cytoskeleton, with deleterious effects on cell viability, organization, and differentiation [3]. HMFs (11–15T) significantly retarded Xenopus laevis development and suppressed gene expression [4]. We were therefore interested in how HMFs may affect cells or animals, how HMFs interact with the body, how humans are affected by HMFs, the health risks associated with HMFs, and how we can apply HMFs to biological systems.

Stable levitation of diamagnetic materials, such as biological macromolecules, cells, tissues, and animal models, has become available using a superconducting magnet with a large gradient of 1360 T^2^/m [5]. Studies in physics, chemistry, materials, and biology using a large-gradient, high-magnetic field (LG-HMF) environment have been carried out in national HMF laboratories, including Japan, Nijmegen, the USA, and France. As diamagnetic objects experience a repulsive magnetic force away from the HMF region, the gravitational force is cancelled by the magnetic field acting on all atoms [6]. If the magnetic field is strong enough, magnetism can affect any atom or molecule. The magnetic body forces (Faraday forces) produced by the gradient magnetic field can therefore be used to simulate different gravity environments, which is one of the most promising tools to realize a virtual microgravity environment on earth. Impressive records of levitating frogs, water drops, insects, mice, plants, and mammalian cells have been reported with high-gradient superconducting magnets [6]–[15]. The LG-HMF imposes a directional ponderomotive force on diamagnetic substances, and thus can simulate gravity or accelerative forces with the advantage that it can be confined to small areas [16], [17]. The LG-HMF also affects various cellular processes and structures and impacts more than simple displacement of amyloplasts [18]. Our previous studies have demonstrated that diamagnetic levitation using superconducting magnets affect the morphology, cytoskeleton architecture, and function of bone cells (osteocytes, osteoblasts, and osteoclasts) [19]–[26] and the development of silkworm eggs [27]. High-gradient magnetic fields can directly affect human pre-osteoclast FLG29.1 cell survival and differentiation [26].

Integrins function as receptors for extracellular matrix (ECM) proteins, such as fibronectin (FN), collagen, and laminin. Collagen I (col I) is the most abundant protein in the ECM of bone, and is required for osteoblastic differentiation. It has been shown that col I expression is decreased in hMSCs (human mesenchymal stem cells) isolated from patients with osteoporosis [28]. The disruption of type I collagen-integrin interactions reduces osteoblastic differentiation [29]. In addition, blocking collagen fibril formation or col I interaction with its integrin receptor (a2β1) *in vitro* decreases ALP expression [30].

In the current study the effects of LG-HMF produced by a superconducting magnet (JMTA-16T50MF) on osteoblast-like cell ultrastructure, ALP secretion, extracellular matrix proteins, and integrin protein expression were investigated. The purpose was to clarify whether or not LG-HMF has fatal effects on mammalian cells, and whether or not the diamagnetic levitation technical platform is suitable for biological studies. The findings of LG-HMF-induced alterations in these characteristics at the cellular level may provide some evidence for the application of diamagnetic levitation technology into biological research, and also improve our understanding of the safety of high-static magnetic fields.

## Results

### The Effects of LG-HMF on Osteoblast-like Cell Viability

**Figure 1:**
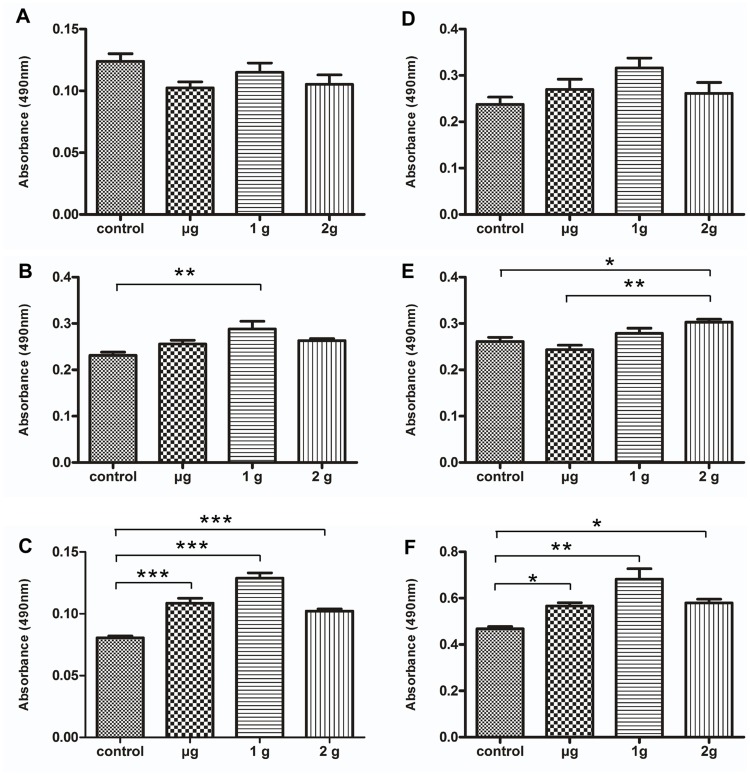
MTT analysis of the effects of LG-HMF on the viability of MC3T3-E1 (A–C) and MG-63 (D–F) cells. After MG-63 and MC3T3-E1 cells were cultured at 37°C for 12 h, 24 h, and 48 h in the bore of the superconducting magnet, the cells were removed from LG-HMF. The MTT assay was used to detect the effects of LG-HMF (μ-g, 1-g, and 2-g) on cell viability. Cell viabilities increased slightly in both cell lines compared to control conditions after 24 h or 48 h of exposure to LG-HMF (*P*<0.05). A–C: MG-63 cells; D–F: MC3T3-E1cells. A, D: exposure to LG-HMF for 12 h; B, E: exposure to LG-HMF for 24 h; C, F: exposure to LG-HMF for 48 h. *: *P*<0.05, **: *P*<0.01, ***: *P*<0.001.

LG-HMF is an extreme man-made environment, thus it is necessary to determine whether or not cells can survive under such a special environment. The MTT cell viability assay showed that LG-HMF (μ-g, 1-g, or 2-g) did not significantly affect the proliferation of MC3T3-E1 and MG-63 cells after 12 h of culturing compared to controls (Fig. 1A and 1D). A moderate increase in the proliferation of MC3T3-E1 and MG-63 cells was shown compared to the control group after 24 h of culturing under LG-HMF (Fig. 1B and 1E). After 48 h of culturing under LG-HMF, the proliferation of MC3T3-E1 and MG-63 cells increased significantly compared with the control group (*P*<0.05, Fig. 1C and 1F).

### The Effects of LG-HMF on Osteoblast-like Cell Ultrastructure

**Figure 2:**
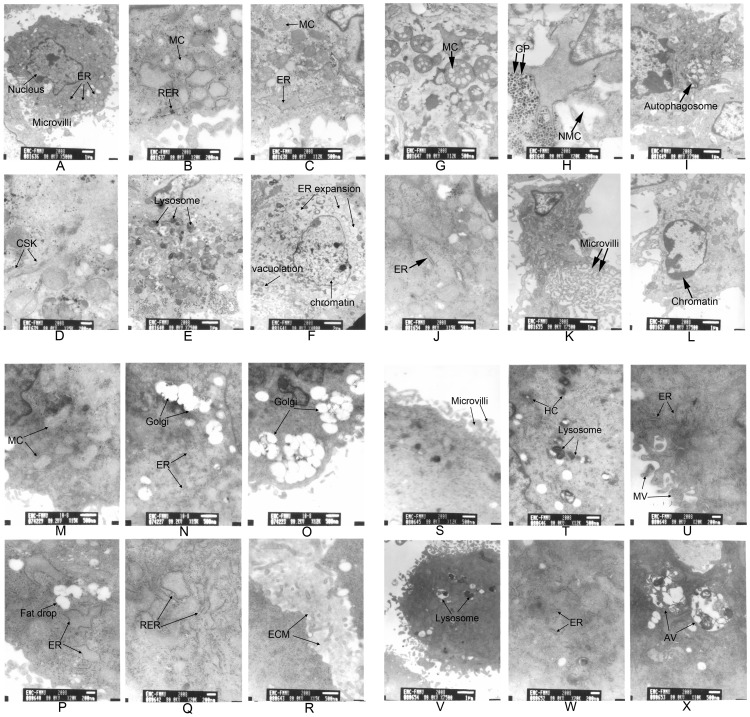
Detection of the effects of LG-HMF on MC3T3-E1 (A–L) and MG-63 (M–X) morphology by transmission electron microscopy (TEM). After MG-63 and MC3T3-E1 cells were cultured in the bore of LG-HMF (μ-g, 1-g, and 2-g) at 37°C for 24 h, the osteoblasts were fixed with 2.5% glutaraldehyde, then dehydrated by gradient acetone and embedded. Ultra-thin sections were contrasted according to Reynolds and examined in a JEOL analytical transmission electron microscope. MC: mitochondria, ER: endoplasmic reticulum, GP: glycogen particles, NMC: nucleus membrane cavity, CSK: cytoskeleton, HC: heterochromatin, MV: microvilli, RER: rough endoplasmic reticulum, ECM: extracellular matrix, AV: autophagic vacuoles. N: A–L: MC3T3-E1cells, M–X: MG-63 cells. A–C and M–O: control, D–F and P–R: μ-g, G–I and S–U: 1-g, J–L and V–X: 2-g.

The effects of LG-HMF on the ultrastructure of MC3T3-E1 and MG-63 cells were investigated using transmission electronic microscopy (TEM). Under normal control conditions, glycogen particles (GPs), rough endoplasmic reticulum (RER), and mitochondria (MC) were abundant and the nucleolus was clear in MC3T3-E1 cells (Fig. 2 A–C, black arrow). In a diamagnetic levitation environment, an increase in the number of lysosomes (Fig. 2D), an abundance of endoplasmic reticulum (ER), aggregation of cytoskeleton (CSK) filaments (Fig. 2E), expansion of the ER, and swelling and vacuolation of mitochondria were noted (Fig. 2F). Swollen mitochondrial cristae, vacuoles, polarity (Fig. 2G), and an excess of heterochromatin (HC), glycogen particles (GP; Fig. 2H), and autophagosomes within some cells (Fig. 2I) were demonstrated under 1-g in a HMF (16 T). Under 2-g in a HMF (12 T), cells had polarity and there were very short ER (Fig. 2J), swelling microvilli (Fig. 2K), and condensed chromatin (Fig. 2L).

Under natural 1 g control conditions, MG-63 cells had good activity, and developed mitochondrial, ER, and Golgi bodies (Fig. 2M–O). Under a diamagnetic levitation environment, significant morphologic changes included abundant mitochondria, primary lysosomes, and RER with little secretory protein (Fig. 2P and 2Q). Secretion of ECM proteins between cells was noted (Fig 2R). Under 1-g with HMF (16 T) conditions, typical changes were characterized by distortion and slight swelling of the distal ends of the microvilli (Fig. 2S). In addition, an excess of heterochromatin (HC), numerous lysosomes and glycogen aggregation, short RER with little secretory protein (Fig. 2T), and cellular polarity were found (Fig. 2 U). Under 2-g with HMF (12 T) conditions, additional lysosomes (Fig. 2 V), short rod-shaped ER with secretory proteins (Fig. 2W), and autophagic vacuoles (Fig 2X) were present.

### The Effects of LG-HMF on Alkaline Phosphatase (ALP) Production in Osteoblast-like Cells

**Figure 3:**
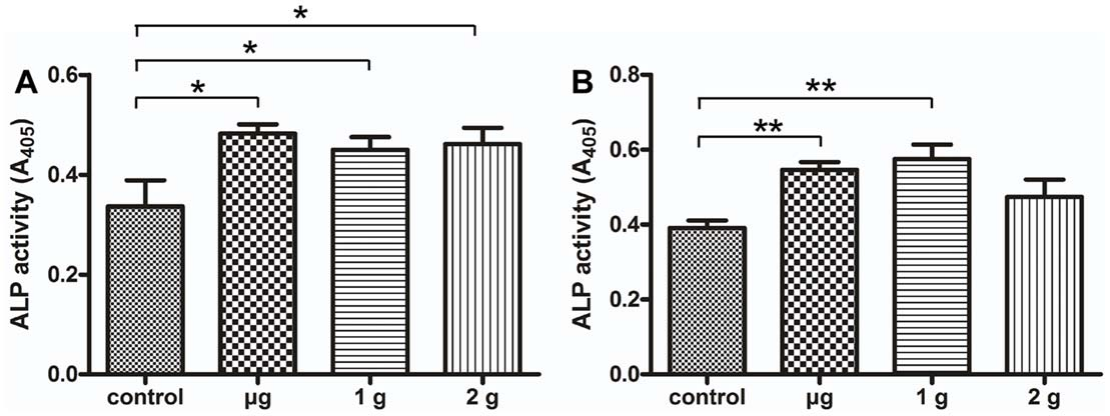
PNPP analysis of the effects of LG-HMF on MC3T3-E1 (A) and MG-63 (B) ALP production. A colorimetric p-nitrophenyl phosphate assay was used to measure ALP expression in osteoblasts after 24 h of exposure to the LG-HMF environment. p-NP was quantified based on the spectrophotometric absorbance at 405 nm. The results indicate that the effects of the magnetic field on ALP were dominant in MC3T3-E1 cells or MG-63 cells. *: *P*<0.05, **: *P*<0.01.

Alkaline phosphatase (ALP), an osteoblast marker, plays an important role in bone formation. To evaluate LG-HMF on bone formation, we examined ALP secretion in MG-63 cells and MC3T3-E1 cells. The results showed that ALP secretion diffusely increased in MC3T3-E1 cells (Fig. 3A) and MG-63 cells (Fig. 3B) cultured under LG-HMF for 48 h (μ-g, 1-g, and 2-g) compared to controls. The difference in ALP production between μ-g, 1-g, and 2-g conditions was not significant (Fig. 3).

### The Effects of LG-HMF on the Expression of Col I and FN in Osteoblast-like Cells

**Figure 4:**
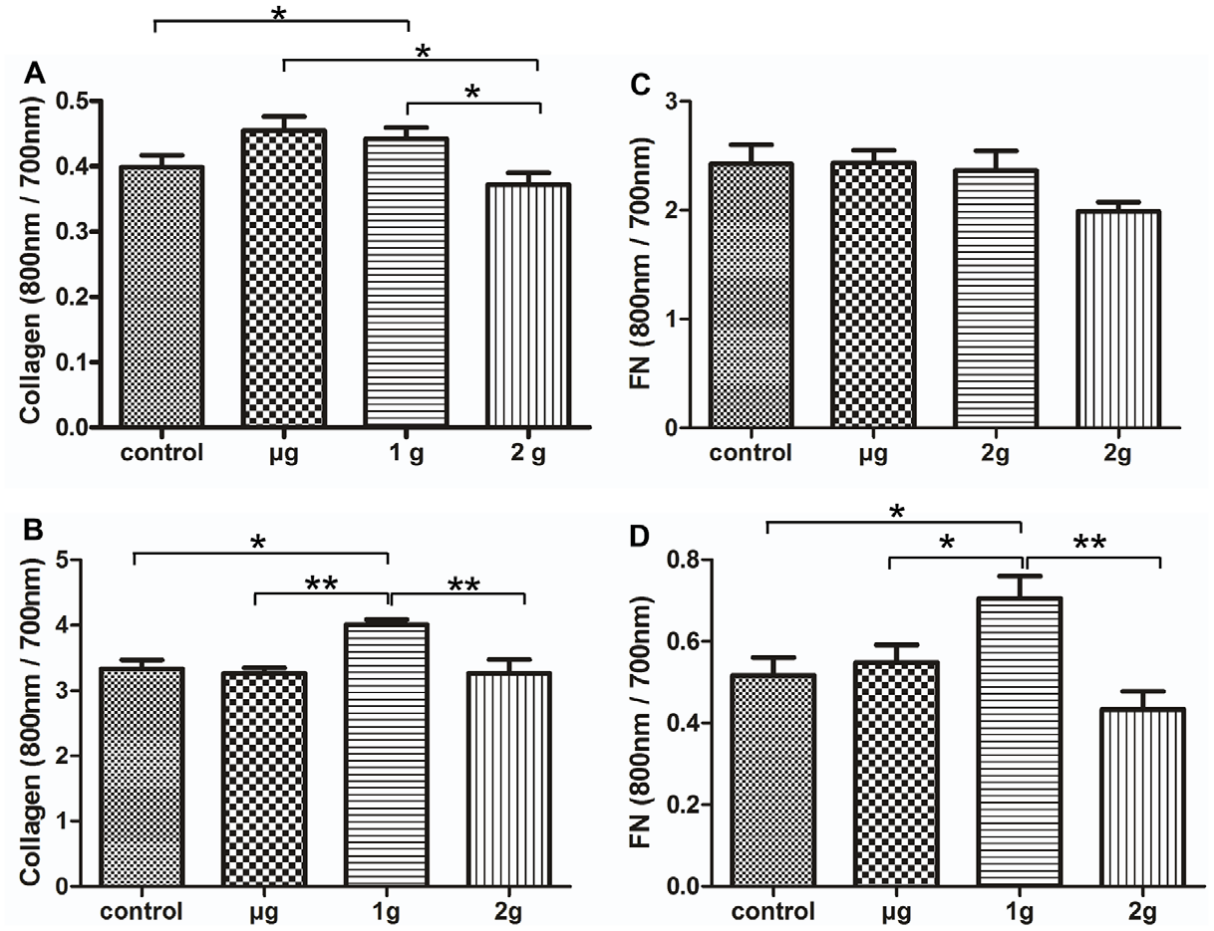
In cell western (ICW) analysis of the effects of LG-HMF on collagen I and fibronectin (FN) expression in MC3T3-E1 (A, B) and MG-63 (C, D). After 24 h of exposure to LG-HMF, the expression of collagen I and FN MG-63 and MC3T3-E1 cells were examined by ICW. The results indicated that collagen I expression significantly increased in MC3T3-E1 cells under μ-g compared to 2-g conditions and 1-g compared to control conditions (*P*<0.05), and in MG-63 cells collagen I expression increased under 1-g compared to control conditions (*P*<0.05). FN expression was also up-regulated in MG-63 cells under 1-g compared to control conditions (*P*<0.05). *: *P*<0.05, **: *P*<0.01.

Type I collagen and FN, as important components of ECM, play an important role in regulating osteoblast differentiation. The expression of col I in MC3T3-E1 cells cultured under μ-g conditions was up-regulated in comparison with 2-g conditions (*P*<0.05, Fig. 4A); however, LG-HMF had no effect on the expression of FN in MC3T3-E1 cells (*P*>0.05, Fig. 4C). A 16.8% and 26.8% increase in the expression of col I and FN occurred in MG-63 cells, respectively, after 24 h of culture in a 1-g with HMF (16 T) environment compared to control conditions (*P*<0.05, Fig. 4B and 4D).

### The Effects of LG-HMF on Expression of Integrin α3, α5, αv, and β1 in Osteoblast-like Cells

**Figure 5:**
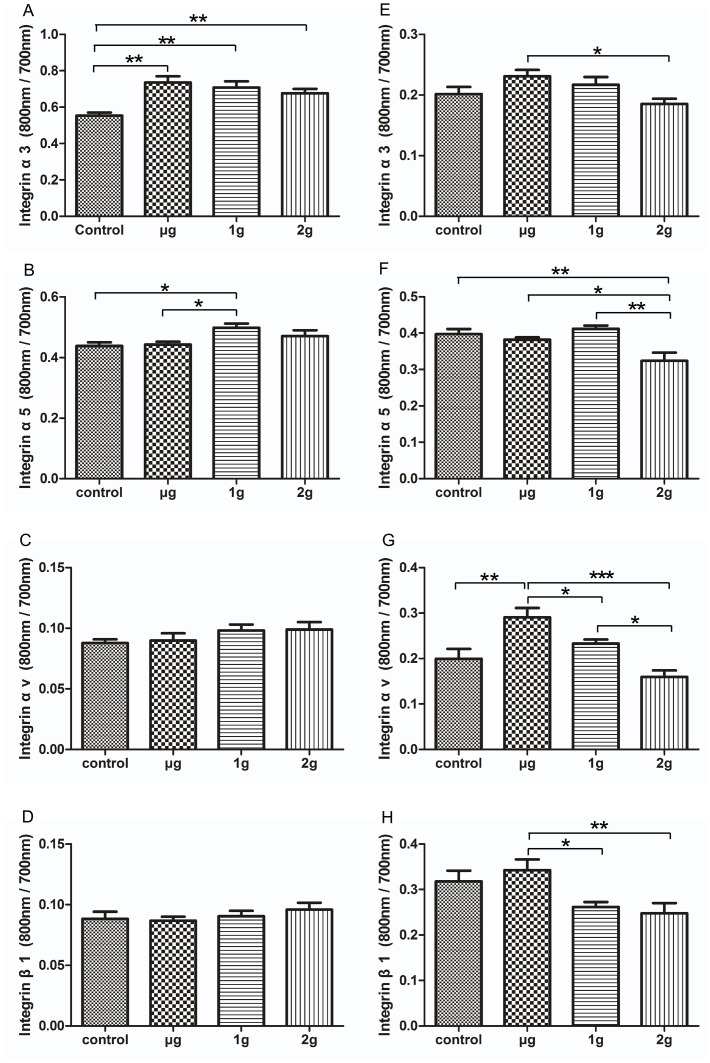
In cell western (ICW) analysis of the effects of LG-HMF on α3, α5, αv, and β1 expression in MC3T3-E1 (A–D) and MG-63 cells (E–H). After 24 h of exposure to LG-HMF, MG-63 and MC3T3-E1 cells were fixed in 4% paraformaldehyde and blocked with blocking buffer. The cells were then incubated with the first antibody, including rabbit polyclonal integrin α3 (H-43) and integrin α5 (H-104), mouse anti-integrin αv, mouse monoclonal anti-integrin β1, and IRDye™ 800-labeled goat anti-rabbit or goat anti-mouse IgG, and DRAQ5. The 96-well plates were scanned simultaneously at 700 nm and 800 nm using an Odyssey Infrared Imaging System. The intensity of each 800 nm value was divided by the corresponding 700 nm value for all wells in the plate. The magnetic field and the reduced gravity worked together to affect integrin protein expression in osteoblast-like cells. *: *P*<0.05, **: *P*<0.01, ***: *P*<0.001.

Integrins, a large family of receptors that attach cells to the ECM, play a critical role in cell adhesion, migration, proliferation, and survival. In the current study, the effects of LG-HMF on the expression of α3, α5, αv, and β1 were detected using a cell western (ICW) assay. The expression of integrin α3 increased in MC3T3-E1 cells under μ-g, 1-g, and 2-g conditions compared with the control group (*P*<0.01, Fig. 5A). The expression of integrin α5 in MC3T3-E1 cells exposed to 1-g conditions was moderately increased compared with control conditions (*P*<0.05, Fig. 5B). The expression of integrin αv in MC3T3-E1 cells exposed to LG-HMF was not significantly different from control conditions (*P*>0.05, Fig. 5C and 5D).

Compared to the 1-g gravity control, LG-HMF (μ-g, 1-g, and 2-g) had no effect on the expression of integrin α3 in MG-63 cells; however, the expression of integrin α3 showed a 21.0% increase compared with μ-g and 2-g conditions (*P*<0.05, Fig. 5E). The expression of integrin α5 in MG-63 cells decreased significantly under 2-g conditions compared with μ-g, 1-g, and control conditions (*P*<0.05, Fig. 5F); the difference between μ-g, 1-g, and controls was not significant. Diamagnetic levitation (μ-g) up-regulated the expression of integrin αv compared with 1-g, 2-g, and control conditions (*P*<0.05, Fig. 5G). The expression of integrin β1 was also increased under μ-g conditions in comparison with 1-g or 2-g conditions (Fig. 5H).

## Discussion

The diamagnetic levitation technique, as a novel technology, has received more and more attention and has been applied in many fields, such as material sciences, biology, and chemistry. In the current study, the effects of diamagnetic levitation on osteoblast ultrastructure and function have been investigated for the first time. The novel and most significant findings of the current study are as follows: the special LG-HMF environment has no lethal effects on osteoblast growth; and a combined environment of HMFs and high-field gradients induces osteoblasts to respond and adapt to the special environment by altering morphology and function.

Normal cell growth and proliferation are the basis for cell-based studies. It is necessary to first determine whether or not cells can survive in an extreme environment. MTT cell viability results showed that the viabilities of MG-63 and MC3T3-E1 cells exposed to LG-HMF for 12 h, 24 h, and 48 h did not decrease, but increased minimally in comparison with controls (Fig 1). LG-HMF does not have a lethal effect on the basic properties of osteoblast growth and survival. Valiron [3] has reported that cell exposure to LG-HMF (>10 T and 15 T in the case of cycling cells and neurons, respectively) affects the cell cytoskeleton with deleterious effects on cell viability, organization, and differentiation. The results indicate that cells from different tissues may respond differently to the external environment.

The endomembrane system of eukaryotic cells includes the ER, the Golgi apparatus, and lysosomes. The membrane lipids and proteins that are synthesized in the ER must be transported via the network to a final destination in membrane-bound vesicles with the help of molecular tags [31]. In the current study, an increase in the number of lysosomes and rough ER, lipid droplets, and dilated ER were observed in electron micrographs after 24 h of exposure to diamagnetic levitation. Cell polarity was clearly established after 24 h of culture under 1-g or 2-g and HMF conditions, suggesting that under diamagnetic levitation, autolytic processes may increase in intensity in cells due to destruction of protein-synthesized organelles. In addition, diamagnetic levitation may affect the process of protein synthesis and transportation. Larger nucleoli and cytoplasm, and a higher volume occupied in the neuronal perikaryon by mitochondriae, ER, Golgi apparatus, lysosomes, and cytoplasmic inclusions (nematosomes) were observed after 15-day-old Sprague–Dawley female rats were flown aboard the space shuttle Columbia (STS-90, Neurolab mission, experiment 150) for 16 days [32]. The morphometry of biosamples (biopsies) of the iliac crest of monkeys flown 14 days aboard the “Bion-11” using electron microscopy showed that some young osteocytes took part in the activation of collagen protein biosynthesis in the adaptive remodeling process of the bone tissue to microgravity conditions. The osteolytic activity in mature osteocytes is intensified and the quantity of empty osteocytic lacunae in the bone tissue increases [33].

The ALPs are a group of enzymes with similar catalytic activity and are expressed in high concentrations in a number of tissues throughout the body. ALP is a marker of bone formation and differentiation due to osteoblast activity [34], [35]. Spaceflight studies have shown decreased serum levels of markers of bone formation, including ALP, osteocalcin, and the C-terminal peptide of pro-collagen type I (col 1a2) [36], [37]. A reduction in markers of osteoblast function, including col I, ALP, and the osteocalcin gene, has been shown in *in vitro* studies of osteosarcoma cells in true microgravity [38], [39]. Our finding showed that 24 h of exposure to LG-HMF (μ-g, 1-g, and 2-g) significantly increased ALP production in osteoblast-like cells compared to controls and the difference was not evident among the μ-g, 1-g, and 2-g groups. These results indicate that a HMF is a dominant factor in inducing ALP secretion of osteoblasts, and the effect of reduced gravity is not clear. A strong static magnetic field (8 T) stimulates bone formation in *in vivo* and *in vitro* systems [40].

Collagen I, as one of the markers of osteoblast function, plays an important role in the process of bone formation. Several studies during space flight have shown decreased serum levels of the C-terminal peptide of pro-collagen type I [36]. Expression of collagen I protein in MG-63 cells significantly increased under 1-g with HMF (16 T) conditions compared to controls (*P*<0.05); expression of collagen I protein was up-regulated in MC3T3-E1 cells in diamagnetic levitation (μ-g) compared to 2-g conditions (*P*<0.05). Our previous study showed that the expression of the collagen I gene in MG-63 and MC3T3-E1 cells was altered by magnetic fields and altered gravity [19]. The results indicate that a HMF is a dominant factor in MG-63 cells, but altered gravity is the determinant in MC3T3-E1 cells. The difference in response between MG-63 and MC3T3-E1 cells may be related to the characteristics of the cells. MG-63 cell line is human osteosarcoma-derived, while MC3T3-E1 is a normal cell line.

FN can be a ligand for dozens of members of the integrin receptor family. Integrins are mechanoreceptors which sense external mechanical stimuli, provide preferred paths for mechanical signals, and transmit these signals across the surface [41]. A large number of different integrins bind to FN, including integrin α5β1, α3β1, and αvβ1. An increase in FN expression in MG-63 cells was detected after 24 h of exposure to 1-g conditions compared to control conditions, but LG-HMF did not affect FN expression in MC3T3-E1 cells. In our previous work the level of transcription of FN in MG-63 cells under diamagnetic levitation conditions was increased significantly compared to other conditions [19] and diamagnetic levitation promoted soluble FN production and 2-g hypergravity inhibited soluble FN production, but LG-HMF did not affect cellular FN expression [42]. Hughes-Fulford [43] has reported that after a total period of 43 h of space flight, osteoblast fibronectin mRNA, protein synthesis, and matrix were unchanged. The expression of integrin α5β1, α3β1, and αvβ1 increased in MG-63 cells under diamagnetic levitation (µg) compared to 2-g conditions (*P*<0.05). The expression of integrin subunits α5 and α3 increased in MC3T3-E1 cells under 1-g conditions compared to control conditions (*P*<0.05); however, the expression of integrin subunits αv and β1 did not change in LG-HMF compared to controls. The integrin β1 subunit is the only binding partner of integrins α3 and α5. Much of the discrepancy between the changes in integrin subunits α3, α5, and β1 may be caused by the composite conditions of the magnetic field and altered gravity, or the resolution in the accuracy of the method of detection. Integrins α5β1, α3β1, and αvβ1 are specific to FN, thus up-regulation of integrins α5β1, α3β1, and αvβ1 may promote FN expression. An alteration in integrin binding ECM proteins (FN and collagen) ultimately leads to an association with cytoskeletal and signaling complexes. Our previous results showed that LG-HMF induced cytoskeleton reorganization in osteoblasts and osteocytes [19]–[23]. This result is similar to a recent report of increased col I-specific α2 and β1 integrin protein expression after 7 days of culture in simulated microgravity [29]. However, decreased expression of integrin αv and β3 subunits have recently been reported after 14 days of hindlimb suspension in a mouse model [44].

In summary, our results show that LG-HMF affects the cellular ultrastructure, ALP production, expression of ECM proteins, and osteoblast expression of integrins α5β1, α3β1 and αvβ1. Moreover, LG-HMF does not have acute lethal effects on osteoblasts. Osteoblasts are sensitive to magnetic fields and altered gravity environments; the underlying mechanism needs to be studied further. The magnetic force environment of high-gradient magnetic fields provides new opportunities for research in diverse areas [10]. HMFs co-exist with apparent gravity levels at all times, thus it is difficult to absolutely distinguish the effects induced by the HMF or by simulated weightlessness. We cannot completely eliminate the possibility of magnetic field effects interfering with reduced gravity simulation, but we can control for it by designing and comparing different experiment groups. Whether or not magnetic field effects interfere with the reduced gravity simulation can be determined by comparison of samples from each of these regions [11]. In any case, diamagnetic levitation, as a fascinating physical phenomenon, has many attractive properties and is worthy of further study.

## Materials and Methods

### Cell Cultures

The mouse osteoblast-like cell line, MC3T3-E1 (China Center for Type Culture Collection [CCTCC], Wuhan, China), and the human osteoblast-like cell line, MG-63 (Cell Collection Center of Shanghai, Shanghai, China), were grown in complete MEM culture medium (Invitrogen Corporation, CA, USA) supplemented with 2 mM L-glutamine, 1.5 g/L of sodium bicarbonate, 0.1 mM non-essential amino acids, 1 mM sodium pyruvate, and 10% fetal bovine serum (FBS; Hyclone, UT, USA) at 37°C in a 5% CO_2_ humidified atmosphere in an incubator for 6 h, then the cells were serum-starved for 12 h. For culturing of cells, 35-mm tissue-culture plates (Nunc, Inc., Roskilde, Denmark) were sealed with parafilm and delivered to the appropriate positions (μ-g, 1-g, and 2-g) in the bore of the superconducting magnet by the object stage. The culture plates were held in position in the magnetic bore by shelves. The monitoring device was integrated into the object stage to measure the gravity, temperature, and displacement. The temperature control system includes a water-bath pump and a channel system and the temperature range for the control system was 37°C ±0.5°C [45].

### Superconducting Magnet

**Figure 6:**
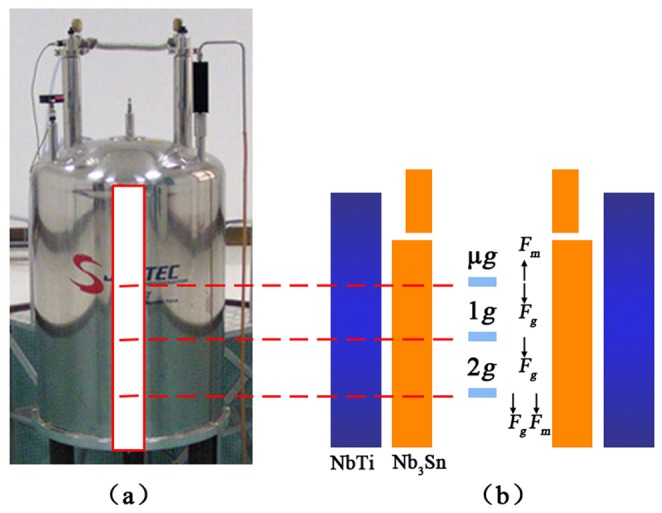
The superconducting magnet with a large-gradient, high magnetic field (LG-HMF) and its specifications. The superconducting magnet can generate a magnetic force field of −1360, 0, and 1312 T^2^/m in a 51 mm (diameter) room-temperature bore, corresponding to 3 apparent body force levels (μ-g, 1-g, and 2-g) and 3 magnetic induction intensities (12 T, 16 T, and 12 T), respectively. The height of the superconducting magnet is 195 cm with a 51 mm × 450 mm (diameter) cylindrical cavity.

An experimental platform for diamagnetic levitation of biological systems has been developed by the authors [45]. Briefly, a superconducting magnet (JMTA-16T50MF; Japan Superconductor Technology, Inc., Tokyo, Japan, Fig. 6), which can provide a LG-HMF, was manufactured by Japan Superconductor Technology, Inc. (JASTEC). The superconducting magnet can generate three different magnetic force fields [*B* ·*(*d*B/*d*z)*] of −1360 T^2^/m, 0 T^2^/m, and 1312 T^2^/m in a 50-mm diameter room temperature bore, corresponding to three apparent body force levels (μ-g, 1-g, and 2-g) and three magnetic induction intensities (12 T, 16 T, and 12 T; Fig. 6). The height of the superconducting magnet is 195 cm, with a 51 mm diameter, 450 mm cylindrical cavity. Increased and decreased gravity environments can be achieved by simply changing the location of the cell sample within the magnet. To develop the biological experiment, equipment matched with the superconducting magnet was designed, including a temperature control system, an object stage, a gas control system, and an observing system [45]. To distinguish gravitational or magnetic field effects, 4 groups were designed in this study (the control group, and geomagnetic fields [1-g, μ-g {12T}, 2-g {12T}, and 1-g {16T}]).

### MTT Cell Viability Assays

To verify whether or not the LG-HMF extreme environment has fatal effects on osteoblast-like cells, the MTT assay was used as previously reported [46]. Briefly, after MG-63 and MC3T3-E1 cells were planted in 96-well plates (9102; Corning Costar, Corning, NY, USA) and cultured at 37°C for 12 h, 24 h, and 48 h in the bore of the superconducting magnet, and the dishes were removed from LG-HMF. Twenty µl of MTT (5 mg/ml; Sigma-Aldrich, St. Louis, MO, USA) solution was then added to 96-well plates (9102; Corning Costar) and cells were incubated for 4 h. MTT solution was removed and 150 µl of DMSO (Pierce Chemical, Rockford, IL, USA) was added. After vibration at room temperature for 20 min to fully dissolve formazan precipitate, the absorbance was detected on a microplate reader at 490 nm.

### Transmission Electron Microscopy

Transmission electron microscopy was used as previously reported [47]. After MG-63 and MC3T3-E1 cells were cultured for 24 h in the bore of the superconducting magnet, the cells were fixed with 2.5% glutaraldehyde in 0.1 M phosphate buffer (PB) for 1 h at 4°C, then thrice-washed with PB. The cells were secondarily fixed for 2 h at room temp in 1% osmium tetroxide in distilled water and twice-washed for 5 min with distilled water. The cells were then dehydrated using 50%, 70%, 90%, and 100% acetone for 15 min. The mixture of acetone and resin (1∶1) was added and mixed on the rotary mixer for 1 h, embedded in a mold, and polymerized in an oven at 60°C for 24 h. Ultra-thin sections were contrasted according to Reynolds and examined in a JEOL analytical transmission electron microscope (JEM-2000EX TEM).

### In Cell Western

An ICW assay was performed according to Odyssey Infrared Imaging System application protocols [42], [48]. After 24 h of culturing, MG-63 and MC3T3-E1 cells were washed and fixed in 4% paraformaldehyde in PBS for 30 min at room temperature, then the cells were permeabilized in 0.1% Triton X-100 for 5 min. After 2 h of blocking with blocking buffer (1% BSA, 5% sucrose, and 0.05% NaN_3_ in PBS), cells were incubated in the following first antibodies in PBS overnight at 4°C: rabbit polyclonal integrin α3 (H-43) and integrin α5 (H-104) (Santa Cruz Biotechnology, Inc., Santa Cruz, CA, USA); mouse anti-integrin αv (BD Pharmingen, San Diego, CA, USA); mouse monoclonal anti-integrin β1 (Calbiochem, San Diego, CA, USA); rabbit polyclonal anti-collagen I (Abcam, Cambridge, UK); and mouse monoclonal anti-fibronectin (Thermo Fisher Scientific, San Jose**,** CA, USA). The specificity of these antibodies was tested using Western blot prior to ICW assay. The cells were then washed 5 times with 0.1% Tween-20 in PBS, and cultured in diluted IRDye™ 800-labeled goat anti-rabbit or goat anti-mouse IgG (1∶2000; LI-COR Biosciences, Superior St. Lincoln, NE, USA) and DRAQ5 (1∶10000; Biostatus Limited, Leicestershire, UK) in Odyssey blocking buffer for 2 h. After all traces of wash solution were removed, the 96-well plates were turned upside down and taped. DRAQ5 is designed to be used in ICW to provide accurate normalization over a broad range of cell densities. The 96-well plates were scanned simultaneously at 700 nm (detection of normalization stains) and 800 nm (detection of IRDye 800CW antibody) using an Odyssey Infrared Imaging System (medium scan quality/169 µm resolution/3.0 mm focus offset/intensity; LI-COR Biosciences). The intensity was divided by each 800 nm value by the corresponding 700 nm value for all wells in the plate. The independent experiments were performed two times.

### ALP Detection

Alkaline phosphatase (ALP) is an enzyme expressed by cells; the activity of ALP has been shown to be a well-defined marker for osteogenesis [49]. A colorimetric p-nitrophenyl phosphate assay was used to measure the expression of ALP in osteoblasts cultured under a LG-HMF environment. After 24 h of culturing under LG-HMF, MG-63 and MC3T3-E1 cells were thrice-washed with PBS and lysed with 0.1% Triton X-100 using 3 cycles of freezing and thawing to verify that the cells were completely lysed. The cell lysates were centrifuged at 15 000 g for 5 min at 4°C, and the supernatant was collected. Fifty µl of cell lysate was transferred to a 96-well plate (Corning) and 150 µl of pNPP (Sigma-Aldrich, Inc.Irvine, UK) was added to each well as substrate for 20 min at 37°C. p-NP was quantified based on the spectrophotometric absorbance at 405 nm. ALP activity was normalized to the total protein concentration for each sample using a BCA protein assay (Pierce Biotechnology).

### Statistical Analysis

Statistically significant differences were determined using one-way ANOVA with Newman-Keuls in Prism statistical software (GraphPad Software, San Diego, CA, USA). A *P<*0.05 was considered significant in all cases.
