# Correction: Large Gradient High Magnetic Fields Affect Osteoblast Ultrastructure and Function by Disrupting Collagen I or Fibronectin/αβ1 Integrin

**DOI:** 10.1371/annotation/6fdbd788-d918-4d42-ac57-fed8323f8508

**Published:** 2013-08-13

**Authors:** Ai-Rong Qian, Xiang Gao, Wei Zhang, Jing-Bao Li, Yang Wang, Sheng-Meng Di, Li-Fang Hu, Peng Shang

A second grant from the National Natural Science Foundation of China was incorrectly omitted from the Funding Statement. The number of the second grant is: 31100667. 

